# Influence of Electrolyte Concentration on Single-Molecule Sensing of Perfluorocarboxylic Acids

**DOI:** 10.3389/fchem.2021.732378

**Published:** 2021-08-03

**Authors:** Xinyun Yao, Ning-Ning Song, Jia Wang, Xian Zhao, Meng-Yuan Cheng, Jiaqi Zuo, Kaipei Qiu

**Affiliations:** ^1^ State Environmental Protection Key Laboratory of Environmental Risk Assessment and Control on Chemical Process, Shanghai, China; ^2^ School of Chemistry and Molecular Engineering, East China University of Science and Technology, Shanghai, China; ^3^ Shanghai Environmental Protection Key Laboratory for Environmental Standard and Risk Management of Chemical Pollutants, School of Resources and Environmental Engineering, East China University of Science and Technology, Shanghai, China; ^4^ Shanghai Institute of Pollution Control and Ecological Security, Shanghai, China

**Keywords:** single-molecule sensing, perfluorocarboxylic acids, electrolyte concentration, nanopore electrochemistry, identification and quantification

## Abstract

Perfluorocarboxylic acids (PFCAs) are an emerging class of persistent organic pollutants. During the fabrication process, it is unavoidable to form PFCA homologs or isomers which exhibit distinct occurrence, bioaccumulation, and toxicity. The precision measurement of PFCAs is therefore of significant importance. However, the existing characterization techniques, such as LC-MS/MS, cannot fully meet the requirement of isomer-specific analysis, largely due to the lack of authentic standards. Single-molecule sensors (SMSs) based on nanopore electrochemistry may be a feasible solution for PFCAs determination, thanks to their ultra-high spatiotemporal resolutions. Hence, as a first step, this work was to elucidate the influence of electrolyte concentration on the four most critical indicators of nanopore measurements, and furthermore, performance of nanopore SMSs. More specifically, three of the most representative short-chain PFCAs, perfluoropentanoic acid (PFPeA), perfluorohexanoic acid (PFHxA) and perfluoroheptanoic acid (PFHpA), were adopted as the target analytes, aerolysin nanopore was employed as the sensing interface, and 2, 3 and 4 M KCl solutions were used as electrolytes. It was found that, when the concentration of KCl solution increased from 2 to 4 M, the conductance of aerolysin nanopore increased almost linearly at a rate of 0.5 nS per molar KCl within the whole voltage range, the current blockade of PFPeA at −50 mV increased from 61.74 to 66.57% owing to the enhanced steric exclusion effect, the maximum dwell time was more than doubled from 14.5 to 31.5 ms, and the barrier limited capture rate increased by 8.3 times from 0.46 to 3.85 Hz. As a result, when using 4 M KCl as the electrolyte, over 90% of the PFPeA, PFHxA and PFHpA were accurately identified from a mixed sample, and the calculated limit of detection of PFPeA reached 320 nM, more than 24 times lower than in 2 M KCl. It was thus clear that tuning the electrolyte concentration was a simple but very effective approach to improve the performance of nanopore SMSs for PFCAs determination.

## Introduction

Per- and polyfluoroalkyl substances (PFASs) are a typical class of persistent organic pollutants (POPs) that are of increasing global concern over the last 2 decades due to their environmental persistence, high bioaccumulation potentials, and associated toxicities (Giesy and Kannan, 2001; Washington et al., 2020). In particular, three of the most widely applied and well-studied PFASs, perfluorooctane sulfonic acid (PFOS), perfluorooctane carboxylic acid (PFOA), and perfluorohexane sulfonic acid (PFHxS), have been listed under the regulation of Stockholm Convention on POPs (UNEP, 2009; UNEP, 2019a; UNEP, 2019b). Recent shift to the short-chain PFAS alternatives (Li et al., 2020) and the structurally modified substitutes (Wang et al., 2017) further caused an enormous growth in the number of PFAS registered in the market, which according to the latest estimate in 2020 has reached 6,330 (USEPA, 2020). The precise detection of legacy and emerging PFASs is therefore of significant importance to understand their occurrence, environmental behavior, and potential health risks (Escher et al., 2020). Despite the lack of national or international standard methods, liquid chromatography-tandem mass spectrometry (LC-MS/MS) is indeed the most common approach to determine priority PFASs given that their authentic standards are readily available (Fiedler et al., 2020). Since 2010, the advances in full-scan high-resolution mass spectrometry (HRMS), including various types of time-of-flight (TOF), Orbitrap, and Fourier transform ion cyclotron resonance (FT-ICR) instruments, have enabled the nontarget identification of a tremendous number of unknown PFASs compounds (Krauss et al., 2010; Liu et al., 2019; Gao et al., 2020). Nevertheless, all the aforementioned methods suffer from high instrument cost and the requirement of highly trained personnel as well as a central laboratory. In this regard, an inexpensive nanopore sensor for the rapid detection of PFASs is highly complementary to the existing analytical techniques and should be focused (Karawdeniya et al., 2019; Menger et al., 2021).

Single-molecule sensors (SMSs) based on nanopore electrochemistry are a unique type of sensors that are able to measure individual analyte molecules one after another (Gooding and Gaus, 2016). By monitoring the through-pore ionic current under a constant potential, nanopore SMSs can link the structures and concentrations of specific targets with the occurrence and frequency of their respective transient current blockade caused by the trapping of single molecules into nanopore (Qiu et al., 2018). Thanks to the ultra-high spatiotemporal resolution, nanopore SMSs enable the accurate identification of rare but crucial species in complex samples (Qiu et al., 2019). The successful commercialization of nanopore based DNA sequencers have also demonstrated the feasibility of developing low-cost, portable nanopore SMSs (Hayden 2015; Quick 2016). During the last 25 years, a wide range of nanopore SMSs have been applied to the determination of metal ions (Braha et al., 2000; Wen et al., 2011), antibiotics (Nestorovich et al., 2002), saccharides (Galenkamp et al., 2018; Ramsay et al., 2018), microRNAs (Wang et al., 2011; Tian et al., 2013; Zhang et al., 2014), proteins (Li et al., 2015; Guo et al., 2018; Liu et al., 2018), but never with PFAS before as far as the authors are aware. Hence, it is worthwhile to explore the possibility of using nanopore SMSs for the effective identification and efficient quantification of PFASs.

Herein, in this particular work, the authors not only presented the concept of PFASs single-molecule sensing, but more importantly, investigated the influence of electrolyte concentration to enhance the performance of nanopore SMS as well. Electrolyte was one of the key components of nanopore systems, and was the simplest variable to be fine-tuned. The effect of electrolyte composition on the four most critical parameters of nanopore detection systems, namely the open-pore current (I_0_) (Smeets et al., 2006), current blockade (∆I/I_0_) (Wang et al., 2019), dwell time (*τ*_on_) (Fologea et al., 2005), and interval time (*τ*_off_) (Franceschini et al., 2016), was reported in separate studies previously. However, only a few of them correlated the values of such indicators to the performance of nanopore SMSs (Fragasso et al., 2020). The following work was thus to elucidate the relationship among electrolyte concentration, four critical indicators of raw current trace, and the performance of nanopore SMSs. Three of the most representative short-chain perfluorocarboxylic acids (PFCAs) were adopted as the target analytes, i.e., perfluoropentanoic acid (PFPeA), perfluorohexanoic acid (PFHxA), and perfluoroheptanoic acid (PFHpA). Aerolysin nanopore was applied as the sensing interface, which featured exceptional resolution to discriminate tiny structural variation, e.g., single amino acid difference (Ouldali et al., 2020). Based on that, the impact of electrolyte concentration was examined in great detail, especially on the qualitative and quantitative analysis capability of nanopore SMSs for PFCAs with different carbon chain lengths.

## Experimental

### Reagents and Chemicals

Potassium chloride (KCl, ≥99.9%), ethylenediaminetetraacetic acid (EDTA, ≥99.0%), tris(hydroxymethyl)aminomethane (Tris, 99%), and octane (≥99%) were purchased from Titan Scientific (Shanghai, China). Aqueous solutions containing 1 mM EDTA, 10 mM Tris, and 2, 3 or 4 M KCl were prepared and used as the different concentrations of electrolytes. 1,2-diphytanoyl-sn-glycero-3-phosphocholine (DPhPC, powder, ≥99%) was purchased from Avanti Polar Lipids (Shanghai, China). Proaerolysin was expressed and purified from Genscript Biotechnology (Nanjing, China). Trypsin agarose (25UN) was purchased from Sigma-Aldrich (Shanghai, China). Perfluorocarboxylic acids and peptide conjugates were produced by ChinaPeptides (Suzhou, China).

### Single-Molecule Sensing of Perfluorocarboxylic Acids

Proaerolysin was activated with the addition of immobilized trypsin at 4°C for 10 h to enable the formation of aerolysin monomers and the subsequent oligomerization for aerolysin nanopore. After activation, the mixture was centrifugated at 10,000 RPM to remove the remaining trypsin and stored at −20°C for the following use. In a typical single-molecule experiment, the temperature of the detection system was kept at 20°C, constantly. A total amount of 150 μl KCl solution of a certain concentration, e.g., 2, 3, or 4 M, was added to the detection chamber. The DPhPC lipid bilayer membrane was formed with the aid of micropipette, spanning the 100 μm microcavitie of MECA 4 Recording Chips (Nanion Technologies, Germany). Aerolysin was then added into the cis chamber. While the cis compartment was defined as the virtual ground, a voltage of +200 mV was applied on the trans compartment to accelerate the insertion of aerolysin nanopore into lipid bilayer. Once a non-zero current change was observed, the external voltage was adjusted to −50 mV to verify the successful formation of a single nanopore. A typical open pore current for a wild type aerolysin nanopore in a 4 M KCl electrolyte was ca. −100 pA at −50 mV. Finally, various types of perfluorocarboxylic acids and peptide conjugates were added into the cis chamber for single-molecule sensing, with a final concentration of 10 μM. As for the recording of electrical signals, the sampling rate and bandwidth were set as 20 and 2 kHz, respectively.

### Data Acquisition and Analysis

The current traces were amplified and recorded using a patch clamp (Orbit mini, together with temperature control unit). The electrical signals were read and classified with Clampfit 10.4. The follow-up data processing and analysis were carried out using laboratory-made scripts based on Python and MATLAB. Graphs were drawn using Origin Pro 2021.

## Results and Discussion

**FIGURE 1:**
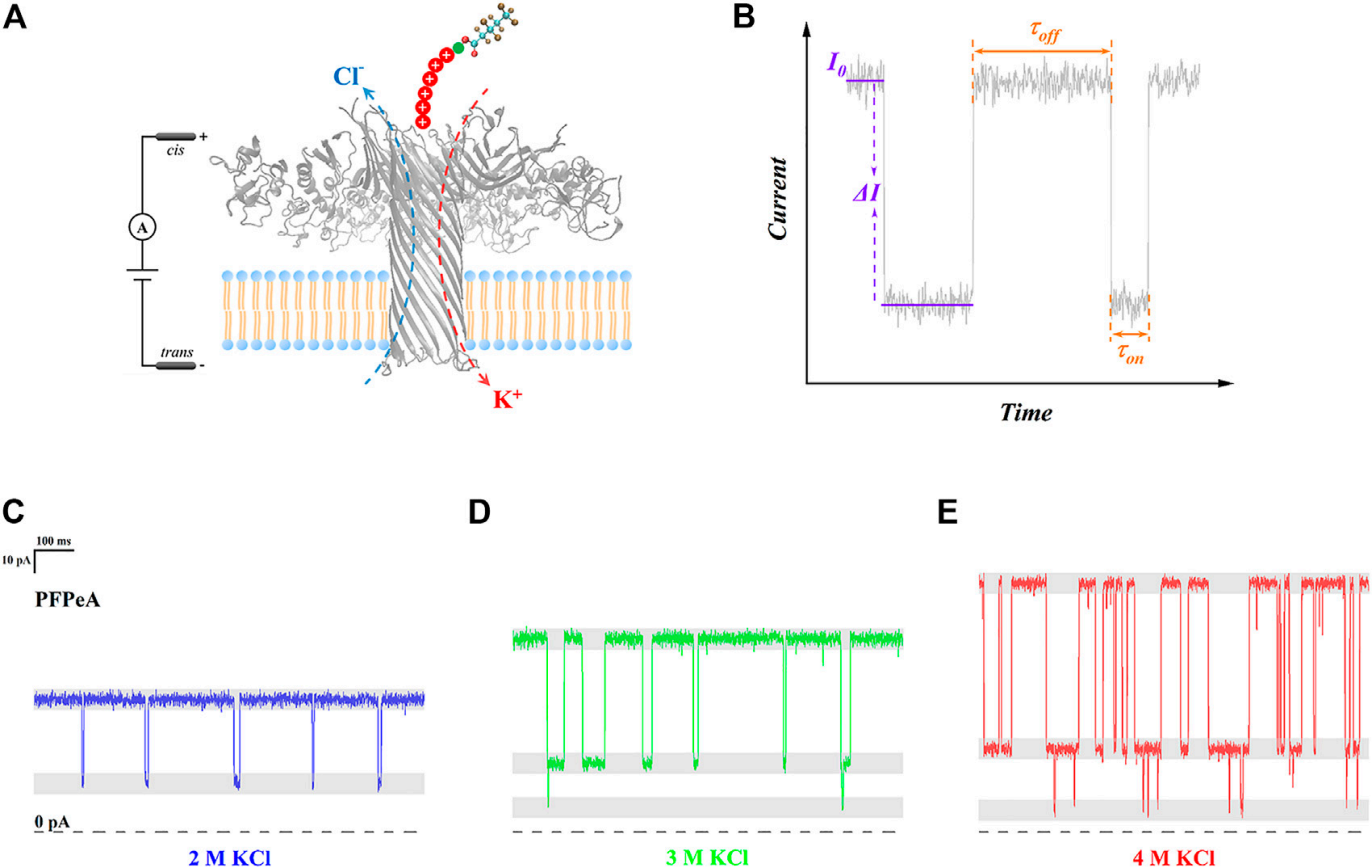
**(A)** Schematic illustration of PFCAs determination by aerolysin nanopore SMSs. **(B)** A typical current trace highlighting the open-pore current (I_0_), blockade (∆I/I_0_), dwell time (*τ*_on_), and interval time (*τ*_off_). Typical current trace of PFPeA detection in **(C)** 2 M KCl, **(D)** 3 M KCl, and **(E)** 4 M KCl electrolytes.

In order to carry out single-molecule sensing of perfluorocarboxylic acids (PFCAs) with nanopore electrochemistry, a unique detection system was rationally designed (Figure 1A): the target PFCA molecule was chemically ligated to a seven-amino-acid-long cationic peptide probe, R_6_K, through a condensation reaction between the amino group at the side chain of lysine and the terminal carboxyl group of PFCAs; while wild-type aerolysin nanopore was embedded into the lipid bilayer, of which the internal diameter was approximately 1 nm, and the total pore length was 10 nm, forming a thin and long β-barrel sensing region (Cao et al., 2016). The role of a positively charged leader was to enhance the capture rate of PFCAs as well as to extend their dwell time inside the nanopore (Willems et al., 2019); or otherwise, no electrical signals could be observed for pristine PFCAs. The prolonged trapping of R_6_K-PFCA conjugates in aerolysin nanopore was due to the combined effect of a strong electrostatic barrier at the trans exit as well as the counterbalanced electrophoretic forces (EPF) and electroosmotic forces (EOF) (Ouldali et al., 2020). In a typical experiment, R_6_K-PFCA analytes were added at the cis side of test chips and were driven to the aerolysin nanopore under a negative voltage.

A typical raw current trace was displayed in Figure 1B, highlighting those four critical indicators. Briefly speaking, here, the open pore current (I_0_) represented the lumen structure and inner charge distribution of aerolysin nanopore, the current change (∆I) was caused by the trapping of R_6_K-PFCA, the dwell time (*τ*_on_) referred to the tendency of a certain molecule staying in the nanopore, and the interval time (*τ*_off_) indicated the capture rate of target analytes. In this sense, the structural characteristics of each individual type of PFCA could be resolved from their respective current blockade (∆I/I_0_), and the value of *τ*_on_, as proposed by the authors, could determine the precision of such identification. With regard to quantification, the magnitude of *τ*_off_ at a given external voltage should be directly related to the limit of detection (LOD). When various concentrations of electrolyte were used, clear differences were found in all four indicators under the same applied bias (Figures 1C–E)—as the concentration of KCl increased from 2 to 4 M, I_0_, ∆I and *τ*_on_ all became greater, while *τ*_off_ was reduced, and the change of ∆I/I_0_ was not obvious. In the meantime, it was also noted that some additional blockade close to 0 pA appeared in 3 and 4 M KCl, and the frequency of their occurrence seemed to increase at a higher concentration of electrolyte. To establish a more quantitative understanding, the influence of electrolyte concentration on those four indicators was systematically analyzed in the next few paragraphs, followed with the illustration of their correlation with the identification and quantification performance of nanopore SMSs for PFCAs.

**FIGURE 2:**
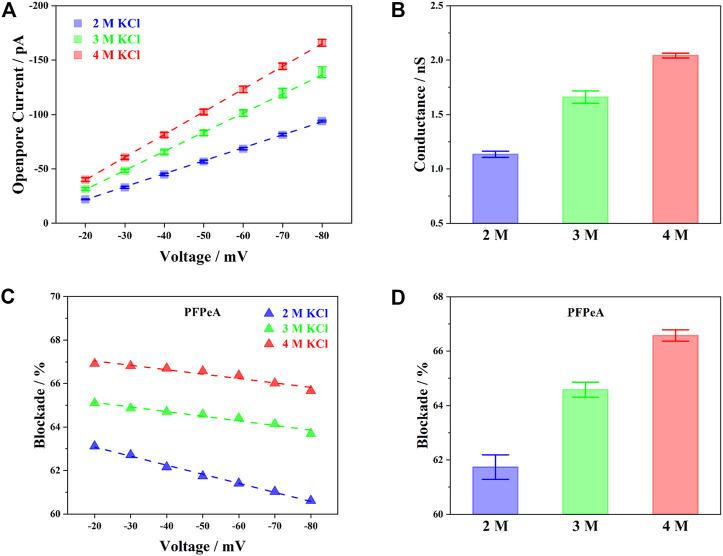
Influence of electrolyte concentration on **(A)** voltage-dependent open-pore current, **(B)** pore conductance at −50 mV, **(C)** voltage-dependent current blockade of PFPeA, and **(D)** the current blockade ay −50 mV.

To start with, the baseline current (I_0_) of aerolysin nanopore in 2, 3 and 4 M KCl was first presented in Figure 2A. The voltage dependent change of I_0_, from −20 to −80 mV, followed a positive linear relationship for all three concentrations of electrolyte, indicating that aerolysin nanopore possessed a constant conductance, and thus stable structure within this voltage range. The more concentrated electrolyte exhibited higher I_0_ at any given potential, and the corresponding conductance at −50 mV was given in Figure 2B. Results showed that the conductance of aerolysin nanopore was almost in proportion to the concentration of electrolyte, ca. 0.5 nS per molar KCl, in accordance with the previous findings for aerolysin nanopore (Cao et al., 2016; Ouldali et al., 2020).

The current blockade (∆I/I_0_), on the other hand, exhibited a distinct voltage-dependent behavior compared to I_0_. Taking perfluoropentanoic acid (PFPeA) as an example, when the external bias increased, the ∆I/I_0_ of PFPeA decreased in all three concentrations (Figure 2C). It was also seen that, at any given voltage, the ∆I/I_0_ in a more concentrated KCl solution was constantly greater than the value in diluted ones. A comparison of the ∆I/I_0_ in three different concentrations of electrolyte at −50 mV was plotted in Figure 2D. It was worth noting that the increase of blockade from 3 to 4 M KCl (1.99%) was actually smaller than the value from 2 to 3 M KCl (2.84%), which was partially in agreement with the greater slope of the voltage induced decrease of ∆I/I_0_ in 2 M KCl. One possible explanation for such phenomena was the reduced Debye length at an elevated concentration of electrolyte (Nomidis et al., 2018). As the salt concentration increased, the contribution of counterion screening was inhibited, and the current blockade became more determined by the steric exclusion effect (Wilson et al., 2019), or in other words, reflecting mainly the structural properties of PFCAs.

**FIGURE 3:**
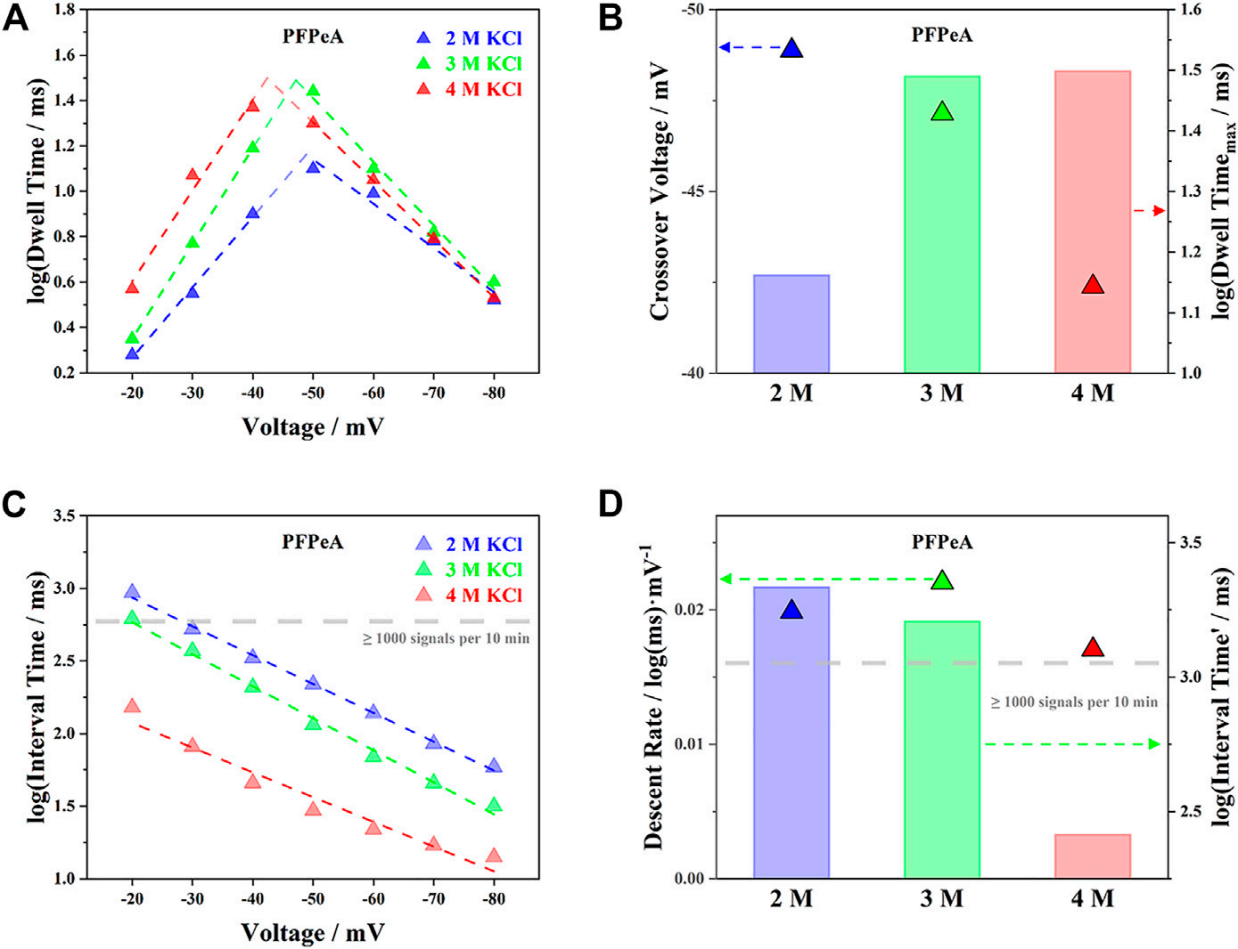
Influence of electrolyte concentration on **(A)** voltage-dependent dwell time of PFPeA, **(B)** cross-over voltage and theoretical maximum dwell time, **(C)** voltage-dependent dwell time of PFPeA, and **(D)** its slope (descent rate) and intercept (interval time at 0 mV).

The trends of dwell time (*τ*_on_) and interval time (*τ*_off_) for PFPeA in 2, 3 and 4 M KCl were summarized in Figure 3, which featured completely different patterns against the applied voltage. As the external bias increased, the logarithm of *τ*_on_, log(*τ*_on_), first rose and then fell when the voltage exceeded −50 mV (Figure 3A), while the logarithm of *τ*_off_, log(*τ*_off_), kept decreasing within the whole range (Figure 3C). Note that, broadly speaking, the total driving force on a single molecule (e.g., the sum of EPF, EOF, etc) to move from the cis entry to the trans exit was enhanced as the electrical potential became higher, it was reasonable to attribute the above observations on *τ*_on_ and *τ*_off_ to the greater energy barrier at the exit than the entrance (Willems et al., 2019). Therefore, at the low potential range from −20 to −40 mV, the total driving force on PFPeA allowed it to enter the aerolysin nanopore from the cis side, but not leave from the trans side—it had to escape back through the cis side, and so a higher driving force from cis to trans led to a longer dwell time inside the nanopore. On the other hand, when the applied bias was over −50 mV, PFPeA was now able to leave through the trans end, and thus the higher driving force corresponded to a smaller dwell time. The linear correlations between the external voltage and the log(*τ*_on_) or log(*τ*_off_) in both ranges further indicated that the above two processes were energy barrier limited (Nomidis et al., 2018).

The threshold for the transition from cis escaping to trans escaping was defined in this work as the crossover voltage, which was estimated by extending the fitting curves of log(*τ*_on_)-voltage trajectories at both low and high potential range (Figure 3A). A higher concentration of electrolyte tended to reduce the magnitude of the respective crossover voltage, as exemplified by the triangle in Figure 3B for PFPeA, since the trans-to-cis EOF was lower, and the overall driving force was enhanced. Besides, the theoretical maximum dwell time reached at the crossover voltage, *τ*_on___max_, followed the trend of 2 M KCl < 3 M KCl < 4 M KCl, which was due to the combined effect of reduced driving force and the balance between energy barriers at both cis and trans side (Ouldali et al., 2020)—the latter was essentially modulated by the interaction between the aerolysin nanopore and R_6_K-PFPeA. The longer dwell time of PFPeA inside the aerolysin nanopore was supposed to improve the precision of SMSs, which would be elaborated in the following identification section.

With regard to *τ*_off_, it was shown in Figure 3C that higher concentrations of electrolyte possessed smaller *τ*_off_ within the whole range of applied voltage. This phenomenon was in alignment with the trend of *τ*_on_ at the low potential range (e.g., −20 to −40 mV), since both of these two processes were determined by the energy barrier at the cis entry of aerolysin. The slopes of the voltage induced decrease of log(*τ*_off_) were comparable in all three concentrations (Figure 3D), while the intercepts of their fitting curves at zero voltage, log(*τ*_off_′), followed the order of 2 M KCl > 3 M KCl > 4 M KCl. Note that the limit of detection (LOD) of nanopore SMSs was defined by both the minimum number of signals for statistical significance and the required response time, a small interval time or a high capture rate of target analytes was essential to approach an excellent quantification capability. To briefly summarize the results so far for *τ*_on_ and *τ*_off_, it was found that as the concentration of KCl solution increased from 2 to 4 M, the theoretical maximum dwell time (*τ*_on___max_) was more than doubled from 14.5 to 31.5 ms, while the entry barrier limited capture rate (1/*τ*_off_′) increased by 8.3 times from 0.46 to 3.85 Hz. In this regard, it was reasonable to argue that higher concentrations of electrolytes offered greater precision for identification and lower LOD for quantification, and in the current study, 4 M KCl was likely to be the most suitable electrolyte of aerolysin nanopore SMSs for PFCAs determination.

**FIGURE 4:**
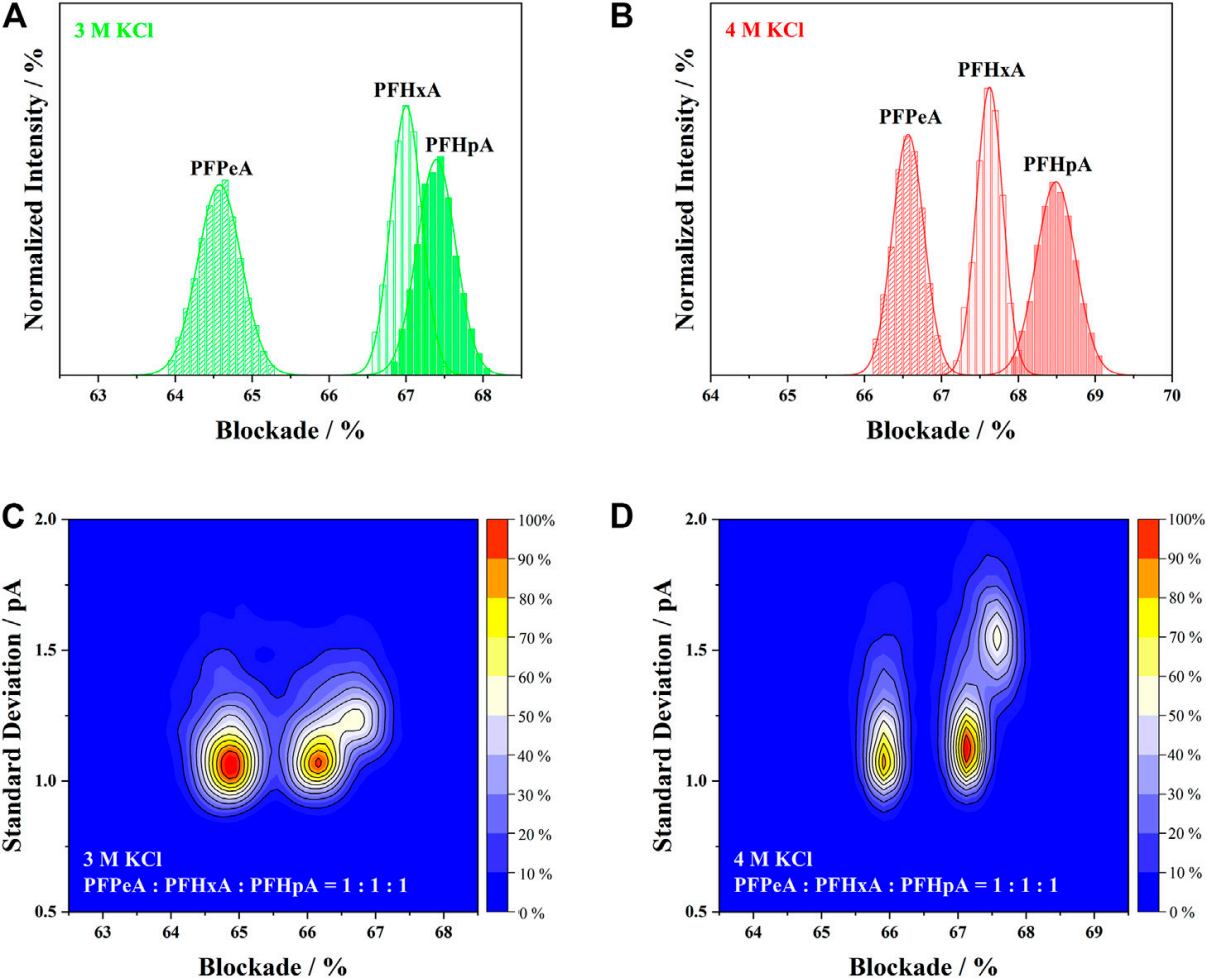
Overlay of the current blockade distribution of PFPeA, PFHxA and PFHpA in **(A)** 3 M KCl and **(B)** 4 M KCl. Discrimination of PFPeA, PFHxA and PFHpA from a 1:1:1 mixed sample in **(C)** 3 M KCl and **(D)** 4 M KCl.

After sorting out the influence of electrolyte concentration on all four indicators, the next step was to elucidate their impact on the performance of nanopore SMSs. The discrimination of structurally similar analytes from mixed sample was crucial for the accurate identification of PFCAs. To compare the effectiveness of aerolysin nanopore in 3 and 4 M KCl, histograms of the current blockades measured separately for PFPeA, PFHxA and PFHpA were overlaid on Figures 4A,B, together with the corresponding gaussian fitting. The area of each fitted curve was normalized, assuming that the total number of signals were the same for all PFCAs, or alternatively, the capture rates were identical. Hence, for those analytes that exhibited greater peak intensity, the respective full-width-half-maximum (FWHM) should be smaller, suggesting a higher precision of the current blockade. It was noted that the FWHM of blockades in 4 M KCl was generally smaller than in the 3 M ones, which was in agreement with the prolonged dwell time caused by the more concentrated electrolytes. Leveraging the concept of resolution in chromatography, the discriminability of nanopore SMSs was characterized by the division of peak separation between two blockades and the sum of their FWHM.

As shown in Figures 4A,B, longer-length PFCAs exhibited higher current blockade in both 3 and 4 M KCl solutions owing to the volume exclusion effect. However, it was evident in Figure 4A that majority of the current blockade of PFHxA and PFHpA in 3 M KCl was overlapped, despite the fact that there was a huge peak separation between the blockade of PFPeA and PFHxA in the same electrolyte. On the contrary, the current blockades of PFPeA, PFHxA and PFHpA were uniformly and well separated in 4 M KCl (Figure 4B). This observation implied the possibility of establishing a structure-blockade correlation for nanopore SMSs (Huang et al., 2019; Ouldali et al., 2020) which would enable the identification of unknown PFASs in the future without using authentic standards. Taking into account the value of peak separation and FWHM, the resolution of PFPeA/PFHxA or PFHxA/PFHpA was calculated to be 1.19 or 0.86, respectively, which indicated that over 90–98% of the analytes could be correctly identified using 4 M KCl as the electrolytes (Zou et al., 2019). Such resolution and precision were at least comparable or even superior to the state-of-the-art performance for nanopore based protein sensors or sequencers (Huang et al., 2019; Ouldali et al., 2020).

The identification capability of aerolysin nanopore SMSs was further evaluated with the mixture of PFPeA/PFHxA/PFHpA in a molar ratio of 1:1:1 (Figures 4C,D). The results were presented in 2D kernel density plots using the magnitude of current blockade as the X-axis and the standard deviation of those blockade as the Y-axis. Three clusters were found in the plots, which according to the current blockade, could be assigned as PFPeA, PFHxA and PFHpA from left to right. The separation of current blockade in the mixed sample measurement seemed smaller than the values obtained from the individual analysis. The corresponding width of current blockade distribution, however, was almost unchanged—taking the 4 M KCl solution as an example, the range of 95% confidence interval indicated that standard deviation of current blockade should be close to or smaller than 0.2 pA for all three analytes. In this sense, it was seen that peak separation of current blockade between PFPeA and PFHxA was comparable in 3 and 4 M KCl, but the distribution of current blockade was much narrower in the latter, which increased the resolution from 80 to 85% to over 95%. Incorporation of standard deviation as an extra dimension for identification was supposed to further enhance the resolution. For instance, it was shown in Figure 4D that the standard deviation of PFHpA in 4 M KCl (1.13 pA) was much higher than that of PFHxA (1.55 pA), and as a result, over 70% of the signals between these two analytes could be resolved, much better than the value of less than 50% in the 3 M KCl counterpart. All the above findings supported that the 4 M KCl electrolyte was more suitable than the 3 M one for PFCAs identification.

**FIGURE 5:**
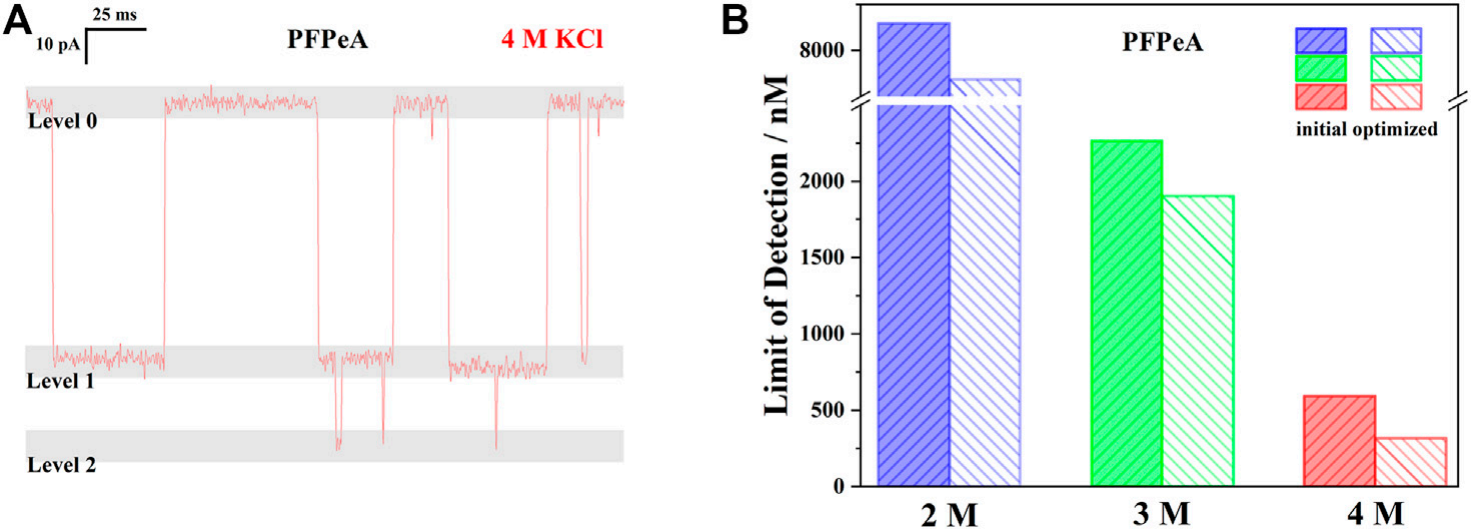
**(A)** A typical current trace of PFPeA in 4 M KCl at -50 mV to highlight the influence of level 2 signal on quantification. **(B)** The initial and optimized limit LOD of PFPeA at −50 mv in 2, 3, and 4 M KCl electrolyte.

Finally, the detection limits of PFPeA in 2, 3, and 4 M KCl were compared to illustrate the influence of electrolyte concentration on nanopore SMSs for PFCAs quantifications (Figure 5). As mentioned, the LOD of nanopore SMSs was actually determined by the minimum number of signals in a given response time. If, for example, at least 1,000 signals were required to be recorded within 10 min, the maximum time allowed for each signal would be 0.6 s on average, including both the dwell and the interval time. Assuming that the dwell time was almost independent to analyte concentration, while the interval time was inversely proportional to it because the concentration gradient decided the diffusion-limited capture processes, it was then possible to quickly estimate the LOD of a certain PFCA based on a single measurement with a known concentration. Meanwhile, the value of *τ*_off_ at a given applied voltage was apparently the key indicator to evaluate the quantification capability of nanopore SMSs. According to the earlier discussion, the interval times of 10 μM PFPeA in 2, 3, and 4 M KCl were 481, 131 and 34 ms, respectively, which corresponded to the LOD values of 8.29, 2.26, 0.59 μM. Therefore, increasing the electrolyte concentration from 2 to 4 M KCl could effectively reduce the LOD by 14-fold (Figure 5B).

Looking more closely to this quantification approach, it was further noticed that the ratio of “signal” to baseline was indeed higher than 1:1, especially in 3 and 4 M KCl, when there were extra blockades close to 0 pA (Figure 5A). To clarify it, the medium range current blockades were defined as the level 1 signals, while those additional greater blockades were called level 2 – and only level 1 signals were used in the previous discussions and calculations. Note that the total number of level 1 signals was equal to the sum of baseline and level 2 signals, based on the event detection principle of Clampfit, an increasing frequency of level 2 thus indicated a higher ratio of level 1 to baseline, and furthermore, reduction of the effective interval time since the contribution of level 2 signals to the total dwell time was negligible, Figure 5A. Following this rationale, the effective LODs of PFPeA in 2, 3, and 4 M KCl electrolyte were re-calculated to be 7.69, 1.90, and 0.32 μM (Figure 5B), and the enhancement from 2 to 4 M was now 24.3 times, more than 70% higher than the initial estimation.

## Conclusion

In summary, the influence of electrolyte concentration on the four indicators of nanopore SMSs and the subsequent performance for the identification and quantification of PFCAs was comprehensively analyzed. When the concentration of KCl solution increased from 2 to 4 M, it was found that the open pore conductance of aerolysin increased almost linearly at the rate of ca. 0.5 nS per molar KCl, the current blockade of PFPeA at -50 mV slightly increased from 61.74 to 66.57% owing to the enhanced steric exclusion effect, the theoretical maximum dwell time was more than doubled from 14.5 to 31.5 ms, and the barrier limited capture rate increased by 8.3 times from 0.46 to 3.85 Hz. As a result, the identification accuracy of PFPeA, PFHxA and PFHpA was significantly enhanced in 4 M KCl, in which over 90% of the analytes was correctly determined from the mixed sample at −50 mV, with the aid of using standard deviation of the current blockade as the extra dimension in 2D kernel density analysis. The effective LOD of PFPeA under the same voltage was estimated to be 320 nM in 4M KCl, which was ca. 24 times lower than the value obtained in 2 M KCl, taking advantage of the higher frequency of level 2 signals in more concentrated electrolytes. From all the above, it was clear that tuning the electrolyte concentration was a simple but very effective approach to improve the performance of nanopore SMSs, both qualitatively and quantitatively. In this regard, our next step was to further examine the influence of electrolyte compositions, e.g., various combination of cations and anions, on PFCAs determination.

## Data Availability

The original contributions presented in the study are included in the article/Supplementary Material, further inquiries can be directed to the corresponding authors.
