# Supplementary material for: Influence of Electrolyte Concentration on Single-Molecule Sensing of Perfluorocarboxylic Acids
Source: Front Chem. 2021 Aug 3;9:732378. doi: 10.3389/fchem.2021.732378 (PMC8369427; doi:10.3389/fchem.2021.732378)
Supplement: Supplementary file 1 [file DataSheet1.DOCX]

Supplementary Material

**Influence of electrolyte concentration on single-molecule sensing of perfluorocarboxylic acids**

**Xinyun Yao^1,2^, Ning-Ning Song^3^, Jia Wang^3^, Xian Zhao^3^, Meng-Yuan Cheng^3^, Jiaqi Zuo^3,*^, Kaipei Qiu^1,3,4,*^**

^1^State Environmental Protection Key Laboratory of Environmental Risk Assessment and Control on Chemical Process, 130 Meilong Road, Shanghai 200237, China

^2^School of Chemistry and Molecular Engineering, East China University of Science and Technology, 130 Meilong Road, Shanghai 200237, China

^3^Shanghai Environmental Protection Key Laboratory for Environmental Standard and Risk Management of Chemical Pollutants, School of Resources and Environmental Engineering, East China University of Science and Technology, 130 Meilong Road, Shanghai 200237, China

^4^Shanghai Institute of Pollution Control and Ecological Security, Shanghai 200092, P.R. China

*** Correspondence:**Jiaqi Zuo
zjq908912128@gmail.com
Kaipei Qiu
kaipeiqiu@gmail.com


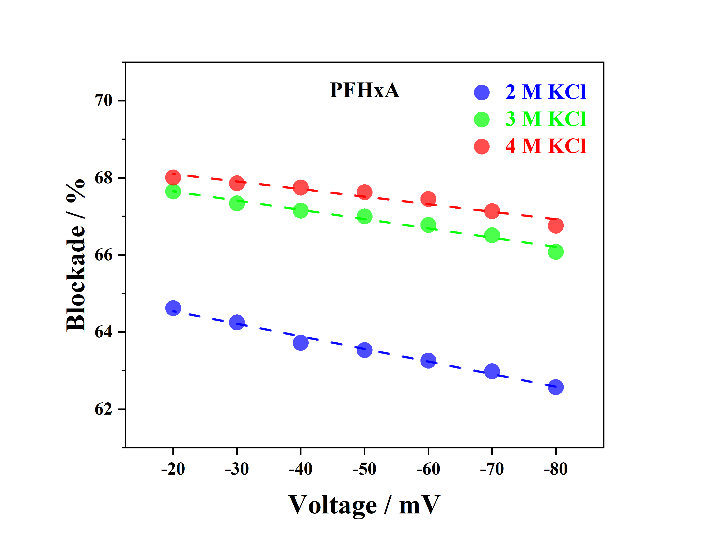

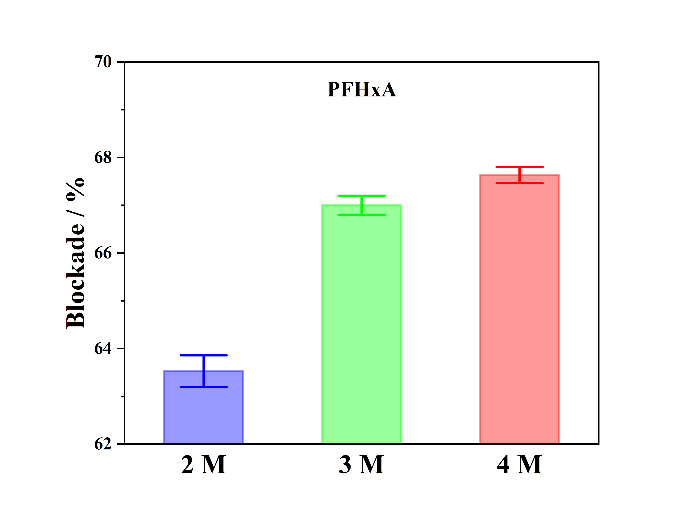


**(b)**

**(a)**

**(d)**

**(c)**


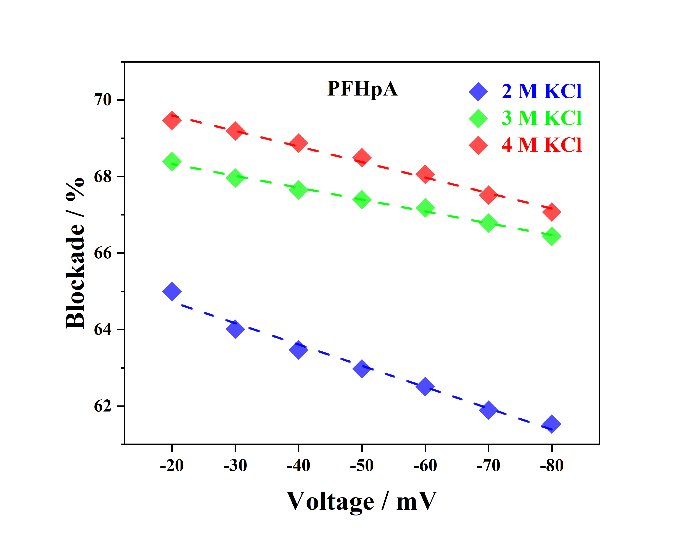

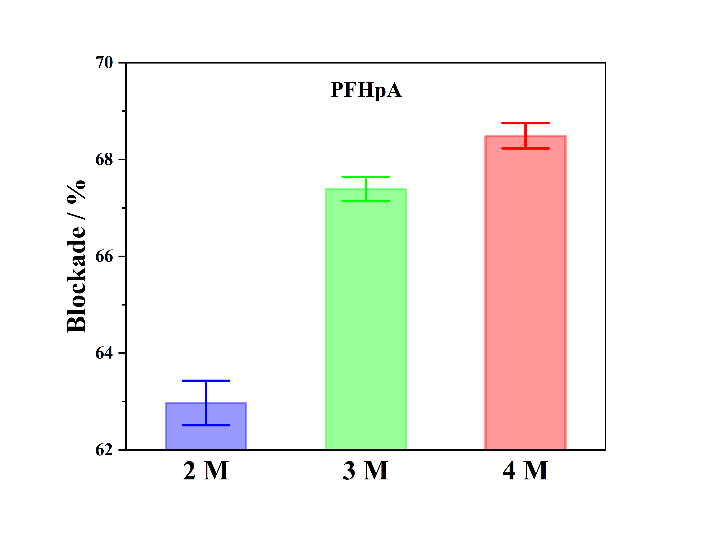


**Supplementary Figure 1**. Influence of electrolyte concentration on voltage-dependent current blockade of (a) PFHxA and (c) PFHpA, as well as the current blockade caused by (b) PFHxA and (d) PFHpA at -50 mV.


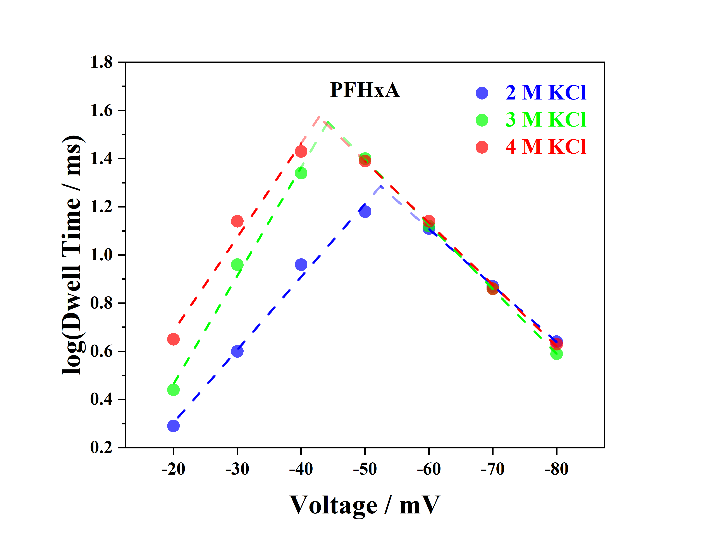

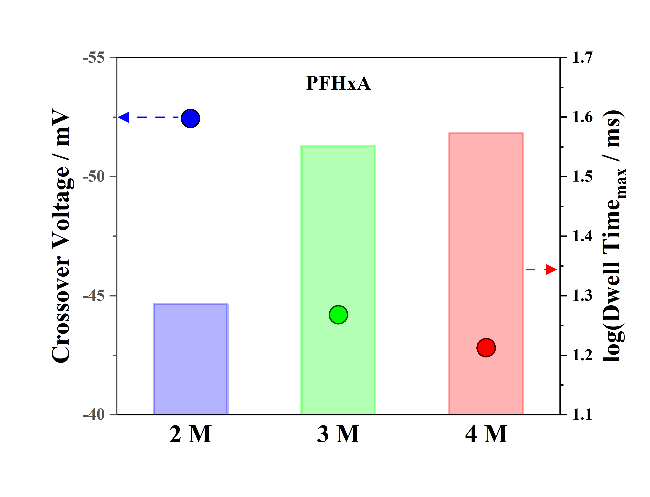


**(d)**

**(c)**

**(b)**

**(a)**


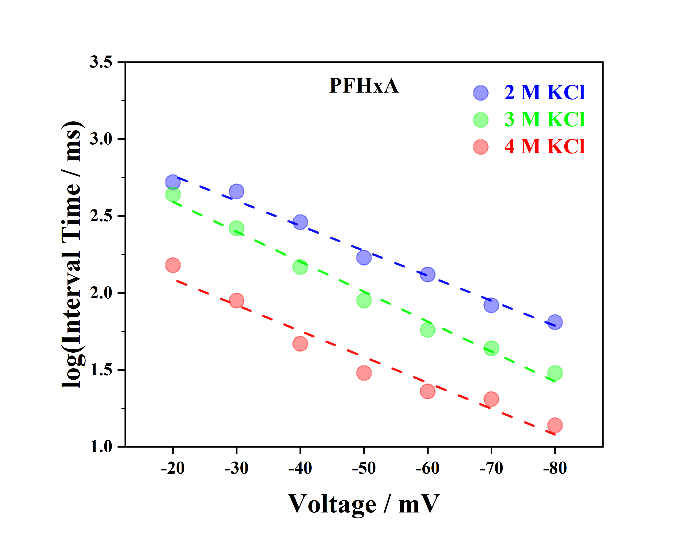

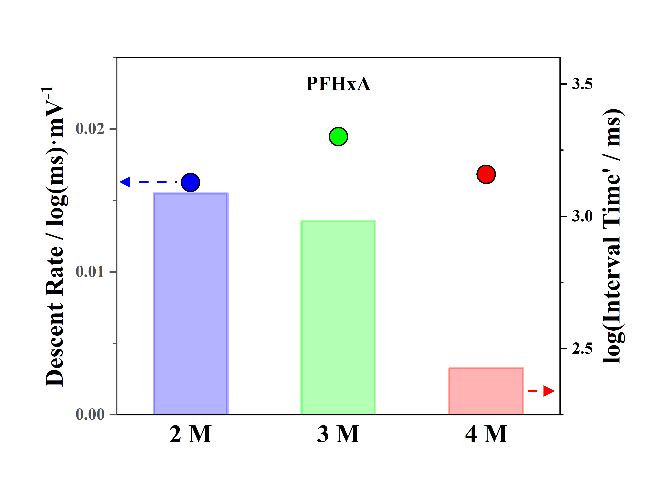


**Supplementary Figure 2**. Influence of electrolyte concentration on (a) voltage-dependent dwell time of PFHxA, (b) cross-over voltage and theoretical maximum dwell time, (c) voltage-dependent interval time of PFHxA, and (d) its slope (descent rate) and intercept (interval time at 0 mV).


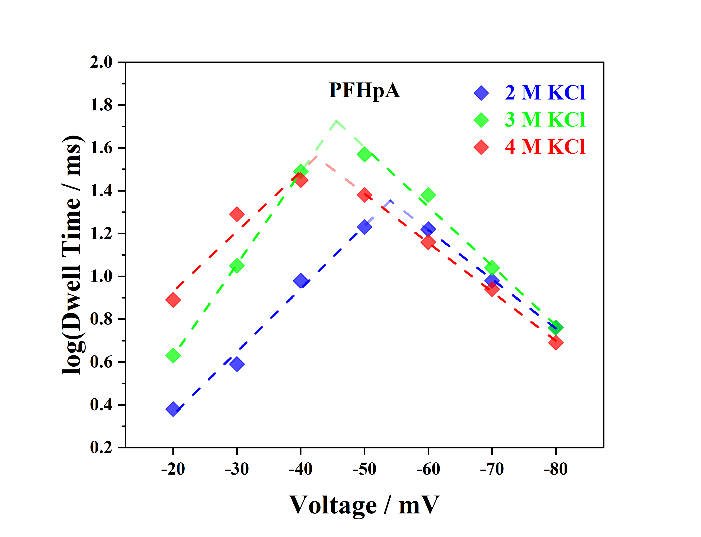

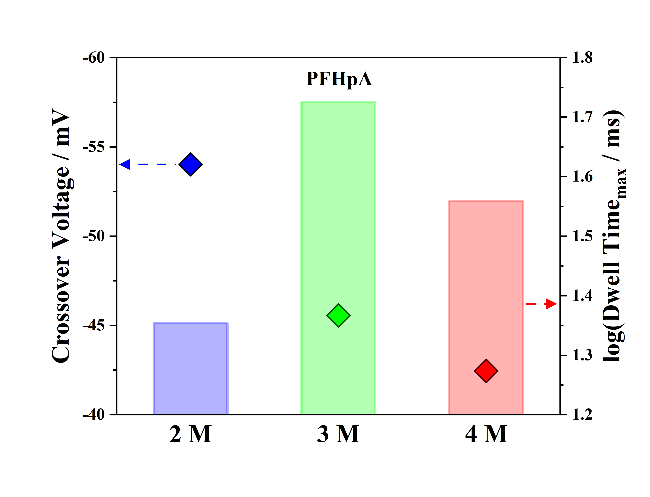


**(d)**

**(c)**

**(b)**

**(a)**


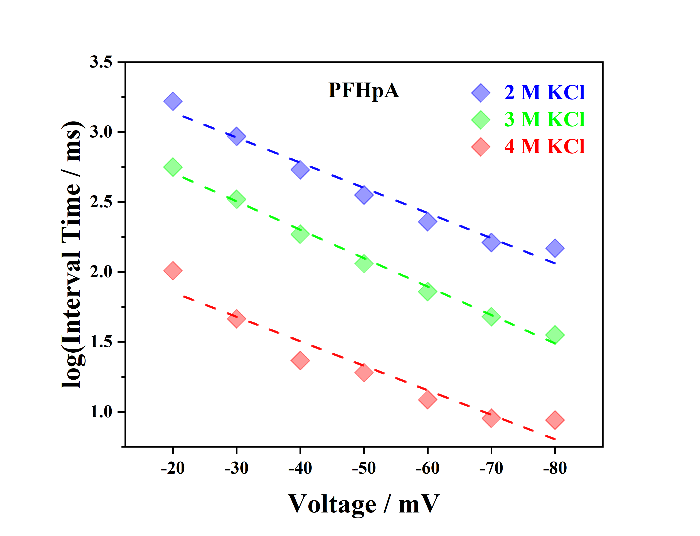

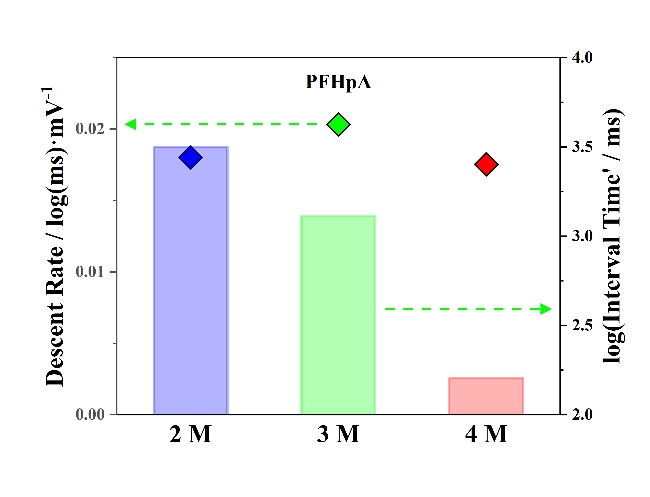


**Supplementary Figure 3**. Influence of electrolyte concentration on (a) voltage-dependent dwell time of PFHpA, (b) cross-over voltage and theoretical maximum dwell time, (c) voltage-dependent interval time of PFHpA, and (d) its slope (descent rate) and intercept (interval time at 0 mV).
